# Modified thoracoplasty for esophago-pleural fistula following Boerhaave syndrome: a case report

**DOI:** 10.1093/jscr/rjaf639

**Published:** 2025-08-25

**Authors:** Qihang Zhu, Jing Zhan, Xiaojing Yao, Haiping Xiao

**Affiliations:** Department of Cardiothoracic Surgery, The First Affiliated Hospital of Guangdong Pharmaceutical University, Guangzhou, Guangdong, China; Department of Cardiothoracic Surgery, The First Affiliated Hospital of Guangdong Pharmaceutical University, Guangzhou, Guangdong, China; Department of Cardiothoracic Surgery, The First Affiliated Hospital of Guangdong Pharmaceutical University, Guangzhou, Guangdong, China; Department of Cardiothoracic Surgery, The First Affiliated Hospital of Guangdong Pharmaceutical University, Guangzhou, Guangdong, China

**Keywords:** Boerhaave syndrome, esophago-pleural fistula, empyema, thoracoplasty

## Abstract

Boerhaave syndrome is a rare but potentially life-threatening condition. Although various optimized treatment methods have contributed to an improvement in the cure rate. However, cases with chronic esophageal pleural fistula and empyema resulting from delayed diagnosis or inadequate treatment occasionally occur. Chronic esophageal pleural fistula and empyema lead to long-term consumption and significant decline in quality of life for patients. We present a case of chronic esophageal-pleural fistula complicated by empyema formation following Boerhaave syndrome, which remained unresolved over a two-year period. A favorable clinical outcome was achieved after the performance of modified thoracoplasty.

## Introduction

Spontaneous esophageal rupture, first comprehensively described by Hermann Boerhaave in 1724, is known as Boerhaave syndrome, typically triggered by severe vomiting after overeating or heavy drinking [1]. As a rare but critical condition, early accurate diagnosis and appropriate management are crucial [2]. Some cases may develop persistent esophagopleural fistulas and localized empyema due to delayed or inappropriate treatment, leading to a refractory course. We present a case report of stepwise management approach, ultimately cured by modified thoracoplasty.

## Case report

A 66-year-old male presented to a local hospital on 15 February 2022, with 1-day chest pain and dyspnea. Unfortunately, spontaneous esophageal rupture was not diagnosed until 4 days later. He underwent left thoracotomy for esophageal fistula repair on 20 February 2022, but the fistula persisted, causing right empyema postoperatively. An esophageal stent was implanted on 18 March 2022, but dislodged and was removed 3 months later. The patient received only enteral nutrition via a duodenal tube and continuous right chest drainage until admitted to our hospital on 7 May 2023, with a 20-kg weight loss.

**Figure 1:**
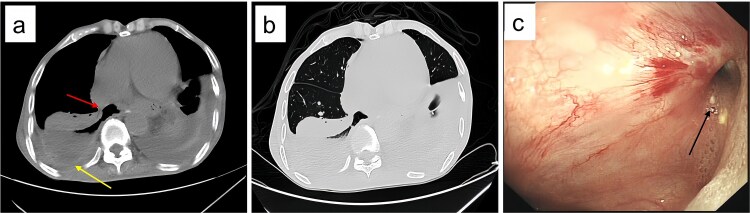
Post-admission chest CT and gastroscopy. (a) Mediastinal window showing esophageal fistula (arrow) and empyema (arrow); (b) lung window confirming the 67 × 36 × 117 mm right empyema; (c) gastroscopy identifying a 8 mm fistula (arrow) 38 cm from incisors.

**Figure 2:**
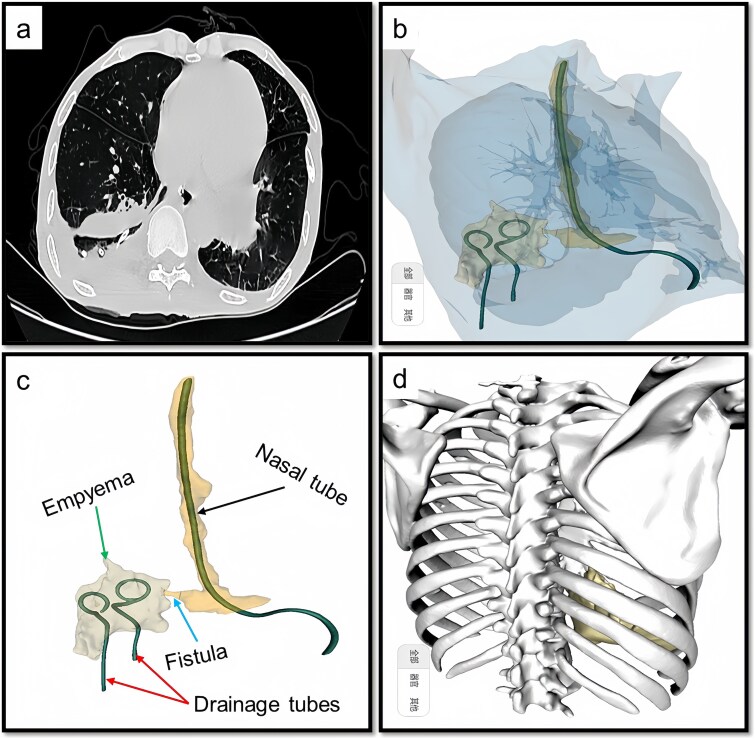
Preoperative CT and 3D reconstruction images. (a) CT revealed that the fistula and associated cavity remained unchanged. (b) 3D reconstruction provides a comprehensive visualization of the fistula and its associated cavity. (c) Demonstration of the details, we can see the fistula (arrow), empyema, nasal tube, and drainage tubes. (d), 3D reconstruction including bones, the 10th rib is situated at the center of the abscess, and accordingly, the extent and number of rib dissections are determined based on this anatomical reference.

**Figure 3:**
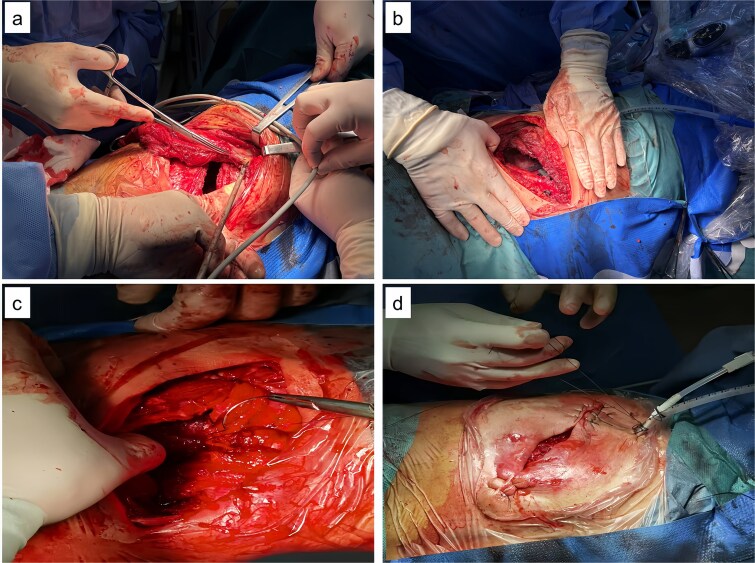
Intraoperative images. (a) Latissimus dorsi muscle flap with preserved blood supply; (b) rib transection and drainage tube placement; (c) muscle flap sutured into the cavity; (d) incision closure.

**Figure 4:**
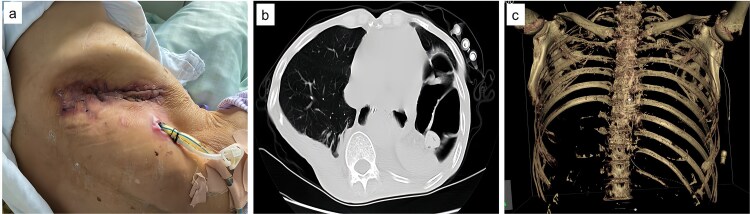
Postoperative images. (a) Fourteen days following the surgical procedure, the wound exhibited satisfactory healing. (b) One month after surgery, during the preparation for removal of the nasointestinal tube, a follow-up chest CT scan revealed that the abscess cavity had been completely filled. (c) Visual documentation of the removal of ribs 9th to 12th.

Upon admission, chest computed tomography (CT) and gastroscopy confirmed persistent esophagopleural fistula and empyema (Fig. 1a-c). A multidisciplinary team formulated a stepwise management plan: Stage 1: Under CT guidance, a chest drainage tube was reinserted. The nutrition team optimized nutritional status, correcting hypoalbuminemia and anemia. Thoracic drainage fluid culture revealed multidrug-resistant *Pseudomonas aeruginosa*; based on antimicrobial committee advice, local treatment (twice-daily 250-ml saline irrigation) was prioritized over systemic antibiotics. Stage 2: After 1 month, the drainage fluid became clearly transparent. However, CT showed unchanged fistula and cavity (Fig. 2a). Thus, a surgical plan was developed. CT-based three-dimensional reconstruction (Fig. 2b-d) guided surgery. Under general anesthesia, a 15-cm incision was made over the 10th rib. The latissimus dorsi muscle flap was dissected with preserved blood supply (Fig. 3a). Intercostal structures were preserved. The 9th and 10th ribs were transected 2 cm from the purulent cavity margin; the 11th and 12th ribs, 2 cm from the vertebral column. After thorough irrigation, a washable drainage tube was placed with its distal end toward the fistula. Muscle flaps were inserted into the cavity and sutured with antibacterial Vicryl (Fig. 3b and c). Finally, the incision was sutured (Fig. 3d). Stage 3: Skin depressions were compressed with cotton pads and a chest strap for 2 weeks (Fig. 4a). Sensitive antibiotics were administered for 2 weeks; intermittent saline irrigation-maintained drainage patency. The drain was removed at 3 weeks. Postoperative CT showed satisfactory recovery (Fig. 4b and c); the duodenal tube was removed 1 month later, and the patient resumed a normal diet gradually. As of July 2025, he remained symptom-free with no recurrence.

## Discussion

Spontaneous esophageal rupture can cause life-threatening infections and multi-organ dysfunction due to leakage of esophageal and gastric contents. Although the incidence of Boerhaave syndrome is relatively low, it continues to be recognized as one of the primary causes of esophageal perforation. Markar *et al*. [3] found 81.9% of 2564 esophageal perforations in the UK (2001–2012) were spontaneous, with 30-day and 90-day mortality of 30% and 38.8%, respectively; recent mortality is 8%–15% [4].

It is generally recommended that surgical repair be performed as early as possible. If surgery is conducted within 24 hours of diagnosis, the mortality rate remains below 10%. In contrast, if the procedure is delayed beyond 24 hours, the mortality rate may increase to 30% [5]. Another retrospective analysis of 88 patients with spontaneous esophageal rupture demonstrated that patients received surgical repair within 24 hours reduced hospital stay, ICU time, and recurrence [6]; Similarly, when surgical repair is delayed for >48 hours after onset, the risks increased [7, 8]. Thoracoscopic minimally invasive repair is now preferred over open surgery. Conservative options include stents or clips, but stents dislodge in 20% (as in this case) and require drainage [9, 10].

Delays in diagnosis or inappropriate treatment may occur. A retrospective analysis of 13 years of clinical experience at a single center in India summarized a total of 12 patients with spontaneous esophageal rupture, of which 10 cases (80%) were diagnosed > 24 hours after symptom onset [11]. Refractory cases with persistent fistulas and chronic empyema require long-term drainage and nutrition, impairing quality of life. In certain cases, digestive tract reconstruction surgery is performed in conjunction with empyema clearance procedures is optional [12]. Due to the reconstruction of the digestive tract and the subsequent alteration of its normal anti-reflux function, patients may experience persistent reflux symptoms following surgery, which can significantly impair their quality of life. Meanwhile, the remnant esophagus may experience subsequent complications, such as the accumulation of esophageal fluid, which could necessitate the long-term placement of drainage tubes or a secondary surgical intervention [13].

Modified thoracoplasty, traditionally for chronic empyema (e.g. tuberculosis) [14], is rarely reported for esophagopleural fistula [15]. In our case, the patient experienced unsuccessful surgical repair and stent placement, accompanied by prolonged parenteral nutritional support. A chronic esophago-pleural fistula with localized empyema subsequently developed. Modified thoracoplasty provides a thorough surgical approach to achieve a cure. Although this case represents a summary of individual case experiences, further validation through multicenter studies and larger sample sizes is necessary. However, given the extremely low incidence rate of spontaneous esophageal rupture, it is challenging to compile a more comprehensive case series within a short timeframe.

## Conclusion

As a classic surgical approach for managing chronic empyema complicated by fistula, modified thoracoplasty demonstrated effective outcome in the treatment of esophago-pleural fistula.

## Data Availability

No new data were created or analyzed in this study. Data sharing is not applicable to this article.

## References

[1] Turner AR, Collier SA, Turner SD. Boerhaave Syndrome. In: StatPearls. Treasure Island (FL): StatPearls Publishing; December 4, 2023. 28613559

[2] Han D, Huang Z, Xiang J, et al. The role of operation in the treatment of Boerhaave's syndrome. Biomed Res Int 2018;2018:8483401. 30050944 10.1155/2018/8483401 PMC6046182

[3] Markar SR, Mackenzie H, Wiggins T, et al. Management and outcomes of esophageal perforation: a national study of 2,564 patients in England. Am J Gastroenterol 2015;110:1559–66. 26437667 10.1038/ajg.2015.304

[4] Aiolfi A, Micheletto G, Guerrazzi G, et al. Minimally invasive surgical management of Boerhaave's syndrome: a narrative literature review. J Thorac Dis 2020;12:4411–7. 32944354 10.21037/jtd-20-1020 PMC7475560

[5] Chirica M, Champault A, Dray X, et al. Esophageal perforations. J Visc Surg 2010;147:e117–28. 20833121 10.1016/j.jviscsurg.2010.08.003

[6] Yan XL, Jing L, Guo LJ, et al. Surgical management of Boerhaave's syndrome with early and delayed diagnosis in adults: a retrospective study of 88 patients. Rev Esp Enferm Dig 2020;112:669–74. 32496118 10.17235/reed.2020.6746/2019

[7] Wang Y, Zhang R, Zhou Y, et al. Our experience on management of Boerhaave's syndrome with late presentation. Dis Esophagus 2009;22:62–7. 18847455 10.1111/j.1442-2050.2008.00858.x

[8] Sutcliffe RP, Forshaw MJ, Datta G, et al. Surgical management of Boerhaave's syndrome in a tertiary oesophagogastric centre. Ann R Coll Surg Engl 2009;91:374–80. 19409144 10.1308/003588409X428298 PMC2758430

[9] Aloreidi K, Patel B, Ridgway T, et al. Non-surgical management of Boerhaave's syndrome: a case series study and review of the literature. Endosc Int Open 2018;6:E92–7. 29344568 10.1055/s-0043-124075 PMC5770272

[10] Hauge T, Kleven OC, Johnson E, et al. Outcome after stenting and debridement for spontaneous esophageal rupture. Scand J Gastroenterol 2018;53:398–402. 29523026 10.1080/00365521.2018.1448886

[11] Surendran S, Victor C, Yacob M, et al. Clinical profile and treatment outcomes of Boerhaave's syndrome: a 13-year experience from an upper gastrointestinal surgical unit. Turk J Surg 2023;39:177–89. 38058370 10.47717/turkjsurg.2023.5830 PMC10696438

[12] Chirica M, Kelly MD, Siboni S, et al. Esophageal emergencies: WSES guidelines. World J Emerg Surg 2019;14:26. 31164915 10.1186/s13017-019-0245-2 PMC6544956

[13] Manickam , Neethirajan S, C SM, Velayoudam V, et al. Giant mucocele of the remnant esophagus: case report of a rare complication following a bipolar esophageal exclusion procedure. Cureus 2019;11:e6317. 31938608 10.7759/cureus.6317 PMC6944150

[14] Kuhtin O, Veith M, Alghanem M, et al. Thoracoplasty-current view on indication and technique. Thorac Cardiovasc Surg 2020;68:331–40. 29772585 10.1055/s-0038-1642633

[15] Mud HJ, van Houten H, Slingerland R, et al. A modified pectoralis muscle flap for closure of postpneumonectomy esophagopleural fistula: technique and results. Ann Thorac Surg 1987;43:359–62. 3566380 10.1016/s0003-4975(10)62802-0

